# Limonin: A Review of Its Pharmacology, Toxicity, and Pharmacokinetics

**DOI:** 10.3390/molecules24203679

**Published:** 2019-10-12

**Authors:** Shunming Fan, Chunling Zhang, Ting Luo, Jiaqi Wang, Yu Tang, Zhimin Chen, Lingying Yu

**Affiliations:** School of Pharmacy, Chengdu University of Traditional Chinese Medicine, Chengdu 611137, China; fanshunming@stu.cdutcm.cn (S.F.); zhangchunling1997@163.com (C.Z.); lt530794033@163.com (T.L.); kikiAPTX@163.com (J.W.); Ty593812828@163.com (Y.T.)

**Keywords:** limonin, pharmacology, toxicology, pharmacokinetics

## Abstract

Limonin is a natural tetracyclic triterpenoid compound, which widely exists in *Euodia rutaecarpa* (Juss.) Benth., *Phellodendron chinense* Schneid., and *Coptis chinensis* Franch. Its extensive pharmacological effects have attracted considerable attention in recent years. However, there is no systematic review focusing on the pharmacology, toxicity, and pharmacokinetics of limonin. Therefore, this review aimed to provide the latest information on the pharmacology, toxicity, and pharmacokinetics of limonin, exploring the therapeutic potential of this compound and looking for ways to improve efficacy and bioavailability. Limonin has a wide spectrum of pharmacological effects, including anti-cancer, anti-inflammatory and analgesic, anti-bacterial and anti-virus, anti-oxidation, liver protection properties. However, limonin has also been shown to lead to hepatotoxicity, renal toxicity, and genetic damage. Moreover, limonin also has complex impacts on hepatic metabolic enzyme. Pharmacokinetic studies have demonstrated that limonin has poor bioavailability, and the reduction, hydrolysis, and methylation are the main metabolic pathways of limonin. We also found that the position and group of the substituents of limonin are key in affecting pharmacological activity and bioavailability. However, some issues still exist, such as the mechanism of antioxidant activity of limonin not being clear. In addition, there are few studies on the toxicity mechanism of limonin, and the effects of limonin concentration on pharmacological effects and toxicity are not clear, and no researchers have reported any ways in which to reduce the toxicity of limonin. Therefore, future research directions include the mechanism of antioxidant activity of limonin, how the concentration of limonin affects pharmacological effects and toxicity, finding ways to reduce the toxicity of limonin, and structural modification of limonin—one of the key methods necessary to enhance pharmacological activity and bioavailability.

## 1. Introduction

### 1.1. General Overview of Limonin

**Figure 1:**
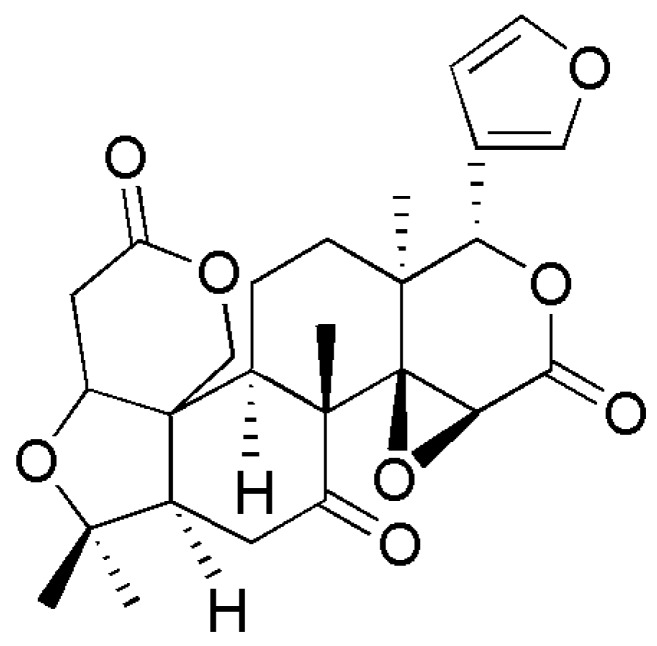
Chemical structure of limonin.

**Figure 2:**
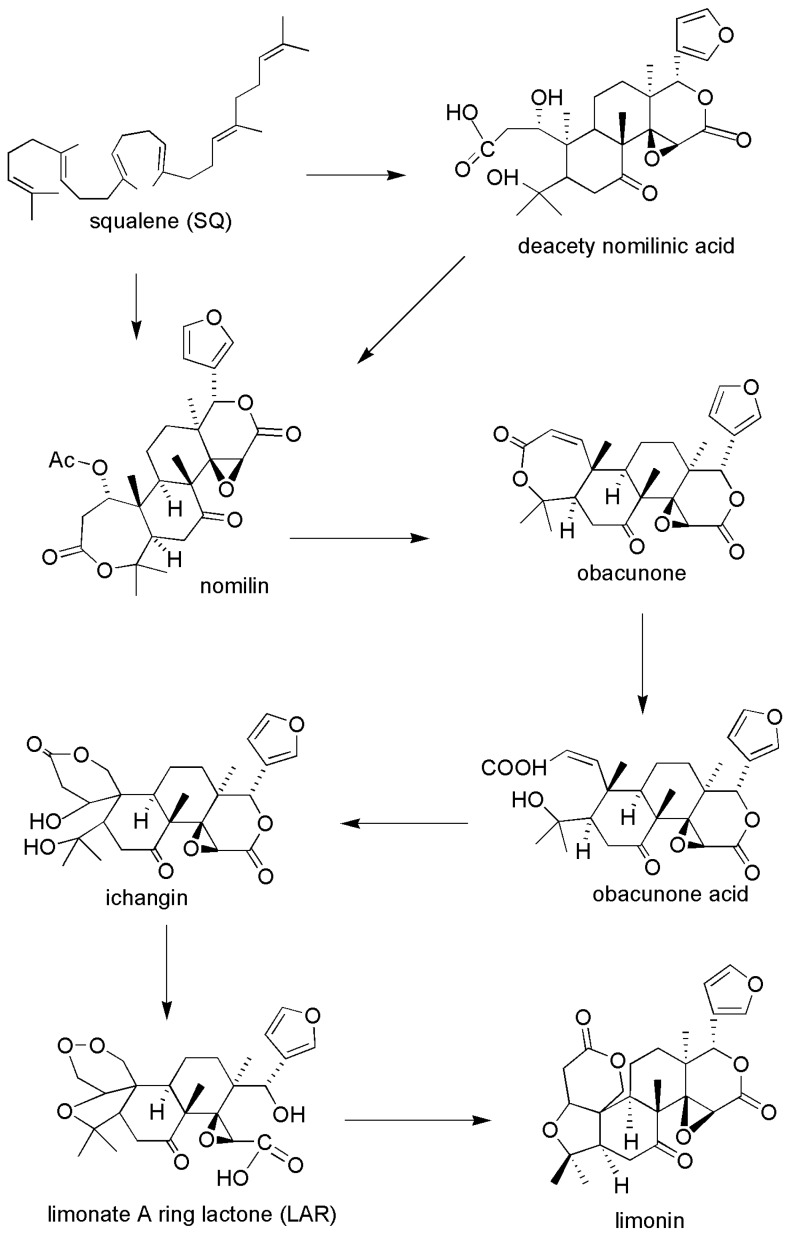
Biosynthetic pathways of limonin in citrus fruit.

Limonin (Figure 1), also known as obaculactone and evodin, belongs to the tetracyclic triterpenoids and is a secondary metabolite with high biological activity in plants. Its molecular formula is C_26_H_30_O_8_ and its molecular weight is 470.25. Limonin is usually derived from the plants of Rutaceae and Meliaceae, and can be isolated from many traditional Chinese medicines (TCM) and fruits (Table 1), mainly including *Evodia rutaecarpa* [1], *Coptidis rhizoma* [2], *Cortex dictamni* [3], *Cortex chinensis phellodendri* [4], bergamot [5], *Aurantii fructus immaturus* [6], *Citri reticulatae pericarpium* [7], and citrus fruits [8], among others. This compound can be found in various parts of plant materials, including fruits, root bark, stem bark, peels, reeds, rhizomes, and roots. Among them, limonin is enriched in citrus fruits and is often found at a high concentration in citrus seeds. In citrus fruits, the biosynthetic pathway of limonin starts from the formation of nomilin by squalene, then limonoids such as obacunone and obacunone acid are obtained by the limonoids pathway, and, finally, the limonoids are metabolized to synthesize limonin. A graphical overview of the main biosynthetic routes involving the limonin in citrus fruit is presented in Figure 2 [9].

At present, the pharmacological effects of limonin are attracting more and more of the attention of researchers. In recent years, many investigations have been done on the pharmacological effects of limonin, and numerous new advances have been acquired. These mainly include anti-tumor, anti-inflammatory and analgesic, anti-bacterial and anti-virus, anti-oxidation, nerve protection, liver protection, and blood lipid regulation. Modern pharmacological effects indicate that limonin has value in the prevention and treatment of certain diseases, including cancer, enteritis, hepatitis, hemorrhoids, osteoporosis, obesity, anaphylactic reaction, and brain aging [13,14].

However, in recent years, the toxicity of limonin has also been reported. Some studies have shown that limonin has hepatorenal and genetic toxicity. Meanwhile, in terms of pharmacokinetics, the poor oral absorption, low bioavailability, and complex effects on liver enzyme metabolism of limonin have also attracted attention. The purpose of this paper is to systematically review and summarize the latest advances in pharmacological effects, toxicity, and pharmacokinetics of limonin. We hope this review can help in exploring the greater medicinal value of this compound and find ways to enhance pharmacological activity and bioavailability.

### 1.2. Materials and Methods

This review paper collected the literature published prior to August 2019 on the pharmacology, toxicity, and pharmacokinetics of limonin. All relevant information on limonin was gathered from worldwide accepted scientific search engines and databases, including Web of Science, PubMed, Elsevier, Wiley Online Library, Europe PMC, ResearchGate, Google Scholar, and Chinese National Knowledge Infrastructure (CNKI). The key words used for the searches were “limonin”, “limonoids”, “phytochemistry”, “toxicology”, “pharmacokinetics”, “pharmacology”, and “activity”. Most of the cited information in this article was from peer-reviewed journals published in English or Chinese. Information was also obtained from PhD and MSc dissertations and from the Chinese Pharmacopeia. No time period limitation was considered in this investigation. Moreover, we did not constrain the studies of limonin contained in plants. Both in vivo and in vitro studies were systematically included in this review.

## 2. Pharmacology

### 2.1. Anticancer Activity

Research showed that limonin has broad and effective anticancer activity. Limonin has certain cytotoxicity to human colon cancer (Caco-2) cells [2]. Limonin can reduce the transcription rate of *BCL2/Bax* and induce the release of cytochrome *C* by activating the endogenous apoptotic pathway. Therefore, limonin can induce mitochondrial-mediated endogenous apoptosis in human colon cancer (SW480) cells [15]. Limonin has been shown to induce cell apoptosis by increasing the expression of proapoptotic protein Bax and decreasing the expression of anti-apoptotic protein Bcl-2 expression in a dose-dependent manner in HCT-15 (liver cancer) and SNU449 (colon cancer) cells [16]. Furthermore, studies have shown that a combination of limonin, limonin glucoside, and curcumin can more effectively inhibit the proliferation of human colon cancer (SW480) cells [17]. It is worth noting that limonin also has potential apoptosis effects on two human cancer cell lines, Caco-2 colonic adenocarcinoma and SH-SY5Y neuroblastoma. Specially, we also found that the effect of limonin glucoside on apoptosis cells becomes stronger than limonin [18]. In vivo, limonin (200 mg/kg) diets inhibited cell proliferation and promoted apoptosis through suppressing the levels of both inducible nitric oxide synthase (iNOS) and cyclooxygenase-2 (COX-2) in azoxymethane (AOM)-injected rats, therefore, it was considered that limonin has the effect of inhibiting colon cancer [19]. In addition, limonin inhibited the proliferation of intestinal cancer cells in tumor suppressor gene (APC)-mutant mice, and decreased the expression levels of proto-oncogene (c-Myc) and monocyte chemotactic protein-1 (MCP-1) mRNA in polyp part [20].

Limonin has an anti-hepatocarcinoma effect. In vitro, limonin inhibited the growth of hepatocellular carcinoma cell line (SMMC-7721) cells (IC50 = 24.42 µg/mL) in a concentration and time-dependent manner [21]. Similarly, limonin can induce apoptosis of human liver-derived hepatoma G2 (HepG2) cells by down-regulating the expression of Wnt signaling pathway lipoprotein receptor-related protein (LRP5, LRP6) and negative regulator of Wnt signaling (DKK) and stabilizing Wnt signaling pathway [22]. Recent research has shown that limonin can inhibit glycolysis of hepatocellular carcinoma cells, thereby inducing apoptosis [23]. In vivo animal experiments have reported that limonin has strong anti-tumor activity—it can resist liver cancer induced by aflatoxin-b1 through the induction of heterogeneous enzymes [24]. In addition, limonin (50 mg/kg) has excellent antioxidant and therapeutic effects on N-nitroethylenediamine (DEN)-induced hepatocarcinoma rats by suppressing lipid peroxidation (LPO) and oxidative stress-mediated free radicals generation, and through modulating antioxidants’ defense mechanism [25].

Research has shown that limonin has an anti-breast cancer effect [26]. It has been reported that limonin has cytotoxic effects on estrogen receptor (ER)-positive (MCF-7) and estrogen receptor (ER)-negative (MDA-MB-231) human breast cancer cells, possibly by activating caspase-7-dependent pathway to achieve inhibition of proliferation activity [27]. In addition, some researchers have also found that limonin can induce apoptosis of breast cancer cell line MDA-MB-231 by inducing ser 468 phosphorylation of nuclear factor kappa-B (NF-κB) pathway to stimulate the expression of apoptotic genes, and considered that limonin to be beneficial in breast cancer patients receiving chemotherapy [28].

In addition, limonin can significantly inhibit the proliferation of human pancreatic islet cancer cell line (Panc-28) cells [29]. It induces cancer cell apoptosis by inhibiting the expression of oncogenes p53 and p21, and activating endogenous pathways such as cytochrome c and caspase-mediated [30]. Moreover, limonin has also been found to induce apoptosis in IOMM-Lee and CH157MN meningioma cells, mainly by inhibiting Wnt5/β-catenin pathway [31]. Aside from this, limonin also has a good antiproliferative effect on lung cancer cells A549 (IC50 = 82.5 uM) [32]. Limonin can also suppress the growth of cervical carcinoma HeLa cells. At the same time, it can inhibit its proliferation and migration [33]. Interestingly, research revealed that limonin has strong inhibitory activity on melanin production in B16 melanoma cells, but has weak cytotoxicity on B16 melanoma cells [34].

Limonin can also inhibit the resistance of cancer cells to anti-cancer drugs. Limonin inhibits the activity of P-glycoprotein (P-gp) in the multidrug-resistant human leukaemia cell line CEM/ADR5000, and inhibits the efflux of the P-gp substrate rhodamine 123 in a concentration-dependent manner. Limonin at the concentration of 20 μM can significantly enhance the cytotoxicity of doxorubicin to drug-resistant CEM/ADR5000 cells [35]. Additionally, limonin can inhibit the stemness of breast cancer cells and attenuate adriamycin resistance in adriamycin-resistant breast cancer cells by suppressing the Wnt/beta-catenin pathway and inhibiting MIR216A methylation [36]. Recent research has found that limonin can attenuate stemness of cervical carcinoma (CC) cells by promoting the nuclear-cytoplasmic translocation of transcriptional coactivator Yes-associated protein (YAP), thus enhancing adriamycin sensitivity and attenuating adriamycin resistance in CC cells [37].

### 2.2. Anti-Inflammatory and Analgesic Activity

The research has shown that limonin can effectively regulate inflammation mediated by CD4^+^ T cells, and inhibit the proliferation of CD4^+^ T cells by suppressing the nuclear translocation of NF-κB P65 in activated CD4^+^ T cells [19,38]. Furthermore, limonin is also involved in the regulation of inflammatory pathway via effectively inhibiting p38 mitogen-activated protein (MAP) kinase activity in vascular smooth muscle cells [39]. Limonin can also offset the metabolic syndrome (MetS)-associated hypertensive and vascular impairment via attenuation of inflammation and fibrosis [40]. Moreover, limonin can effectively inhibit the excessive production of NO in RAW264.7 macrophages activated by lipopolysaccharide (IC50 = 231.4 μM) [41]. Further research discovered that limonin inhibited the production of NO by suppressing iNOS gene expression through NF-κB mediated pathway [42]. Recent studies have shown that limonin has an excellent therapeutic effect on trinitrobenzene sulfonic acid (TNBS)-induced inflammatory bowel disease (IBD), and its efficacy in reducing pathological damage may be comparable to mesalazine [43]. In addition, limonin alleviated dextran sulfate sodium (DSS)-induced colonic inflammation in mice by inhibiting the activation of NF-κB p65, and inhibiting inflammatory cell infiltration and intestinal mucosal necrosis [44]. Limonin significantly decreased the levels of tumor necrosis factor-α (TNF-α), interleukin (IL-1β and IL-6), and inhibited the expression of inflammatory factors in lipopolysaccharide (LPS)-induced acute lung injury mice [45]. For chronic airway inflammation rats, limonin can inhibit the production of mucin by activating the expression of bitter taste receptor gene (TAS2Rs) in the lung [46]. In addition, limonin has a good therapeutic effect on contact hypersensitivity dermatitis, including decreasing the mRNA expression of IL-2, IL-17a, TNF-α, and interferon γ (IFN-γ) in lymph nodes of mice with dermatitis in a dose-dependent manner, and suppressing growth in activated T lymphocytes to against the contact hypersensitivity [47]. Meanwhile, limonin also has good preventive and therapeutic effects on non-bacterial prostatitis (NBP) by regulating metabolic pathways [48]. Limonin has good anti-inflammatory activity on carrageenan-induced paw edema and collagen-induced arthritis in rats [49]. However, its specific mechanism demands further research.

A recent study has found that a novel water-soluble derivative of limonin (3C) is synthesized via the oximation at the C(7)-position of limonin and subsequent introduction of tertiary amino group (R = -CH_2_CH_2_N(CH_2_CH_3_)_2_) by etherification of limonin oxime, which has stronger anti-inflammatory and analgesic activities [50]. Moreover, subsequent research had found that 4-atom (-NH-CO-CH_2_-CH_2_-)-length linker between C(7)-position and tertiary nitrogen atom and the oxygen bridge between C(14) and C(15) of limonin were beneficial in their anti-inflammatory activity. Therefore, it was considered that the water-soluble derivatives of limonin modified by structure could play a better anti-inflammatory effect [51,52]. In addition, limonin glucoside can decrease several circulating markers of hepatic inflammation, and effectively reduce cell proliferation and inflammation [53]. Therefore, structural modification of limonin is an important direction to follow in order to enhance its anti-inflammatory and analgesic activities in the future.

### 2.3. Antibacterial, Antiviral, and Anti-Insect Activities

Studies have shown that limonin has an effective bacteriostatic effect on *Escherichia coli*, *Staphylococcus aureus*, *Bacillus thuringiensis*, *Bacillus cereus*, *Salmonella*, *Micrococcus luteus,* and *Shigella* spp., and also has great bacteriostasis on fungi [54,55]. However, the bacteriostatic effect of limonin on *Mucor* is not obvious, and reports indicated that the bacteriostatic ability of limonin on other bacteria is as follows: *Bacillus subtilis* > *Staphylococcus aureus* > *Escherichia coli* > *Aspergillus niger* > *Shigella* > *Salmonella* [56]. Furthermore, in vitro bacteriostasis experiments showed that limonin had an effective antibacterial effect (Minimum inhibitory concentration (MIC): 15.62~62.5 µg/mL) against *Xanthomonas* spp., and limonin can cause the morphological changes of *Xanthomonas* sp. SK12 at the MIC (15.62 µg/mL) [57]. Limonin has a significant antibacterial effect against *Acinetobacter baumannii*. Computational screening suggested that limonin displays promising binding potential towards diaminopimelate epimerase (DapF) and uridine dipho-sphate (UDP)-N-acetylglucosamine 1-carboxyvinyltransferase (MurA) of *A*. *baumannii*. Meanwhile, the molecular dynamics simulation also validates the docking results [58]. In addition, it has also been reported that limonin interferes with the transmission of signals between bacterial cells [59]. For example, limonin can effectively inhibit intercellular communication, biofilm formation, and type three secretion system (TTSS) of enterohemorrhagic *Escherichia coli* (EHEC) via a quorum sensing-dependent fashion [60]. Subsequent research found that the furan ring and C-7 substitutions had important structural features in limonin molecule, which could promote the inhibition of cell–cell signaling and biofilm formation in *Vibrio harveyi*. Moreover, the modification of limonin (limonin 7-methoxyoxime) inhibited *E. coli* biofilm activity through the interaction of type 1 pili and Ag43 with an IC25 of 53.7 μM [61]. Natural limonin has an effective antibacterial effect, but the relevant dose of limonin derivative has stronger antibacterial activity than natural products. Modification in A-ring and at C-7 position of the limonin structure generated compounds that had strong antimicrobial activity [62].

Limonin can effectively inhibit the replication of human immunodeficiency virus-1 (HIV-1) (EC50 = 60.0 μM) in a dose-dependent manner, and its mechanism may be related to the inhibition of HIV-1 protease activity [63]. In addition, research has reported that the concentration of limonin at 100 µg/mL inhibited the infectious activity of herpes simplex virus (HSV) type 1 and 2 [64]. Limonin can inhibit human T-lymphotropic virus 1 (HTLV-1) tax/rex expression at the concentration of 5 µg/mL. Meanwhile, the HIV-1 gag expression was completely inhibited by limonin with a concentration of 1 µg/mL. Hence, limonin had effective antiretroviral activity against HTLV-1 and HIV-1 infection in vitro [65].

Limonin exhibited good antimalarial activity against new ring-stage *Plasmodium falciparum* with IC50 values of 2.7 μM [66]. Moreover, limonin also possessed potent nematocidal activity with IC50 values of 197.37 µg/mL [67]. Other studies found that limonin had effective bioactivity against *Schistosoma mansoni* in juvenile and adult stages, and its anti-parasitic activity was enhanced in a dose-dependent manner [68]. However, the specific mechanisms of limonin against schistosomes requires further study.

### 2.4. Antioxidant Activity

The study found that the limonin extracted from the tissues of buntan (*Citrus grandis* Osbeck) fruit had strong antioxidant activity [11]. Similarly, the limonin isolated from citrus peel alcohol extract also revealed strong determining radical scavenging activity (DPPH) free radical scavenging activity, which possessed good antioxidant activity [69,70]. In addition, the antioxidant activity of limonin was determined by β-carotene bleaching assay. It was found that limonin has excellent antioxidant activity, even better than vitamin C [71]. The purified limonin from red Mexican grapefruit showed effective antioxidant activity by determining radical scavenging activity (DPPH) and total phenolic content [72]. Moreover, limonin not only inhibited the generation of oxygen free radicals in superoxide model, but also reduced the accumulation of fatty acid oxidation products in hamster low-density lipoprotein (LDL). It was also believed that the weak oxidation activity of highly oxidized triterpenoids was owing to less hydroxyl groups in the structure and poor water solubility [73]. In natural aging rats, limonin can decrease the levels of malonaldehyde (MDA) and lipofuscin in serum and brain tissue, increase the activity of superoxide dismutase (SOD) and glutathione peroxidase (GSH-Px) in serum and brain tissue, and enhance the ability of total antioxidant (T-AOC) in brain tissue [74]. Remarkably, the limonin glucoside after structural modification of limonin can also achieve antioxidant activity by scavenging free radicals [75]. However, some researchers also questioned whether limonin has antioxidant activity [76]. At present, there is still a lack of research on the mechanism of antioxidant activity of limonin, which is worth exploring for this natural antioxidant in the future.

### 2.5. Liver Protection Activity

Research showed that limonin possessed an inhibitory effect on cytochrome P450 3A (CYP3A) [77]. Moreover, limonin caused a dramatic decrease in residual CYP3A4 activity, thereby suppressing cytochrome P450 3A4 (cyp3A4)-mediated human liver microsomal erythromycin N-demethylation activity. Therefore, it was considered that limonin has a certain hepatoprotective activity [78]. Meanwhile, limonin exhibited a protective effect on hepatic ischemia reperfusion (I/R) liver injury rat, whose mechanism was related to the down-regulation of Toll-like receptor (TLR)-signaling transduction pathway [79]. The study found that pre-administration of limonin can significantly attenuate marker of hepatic damage (elevated liver enzyme activities), hepatic inflammation (TNF-α, neutrophil infiltration), oxidative stress and the expression of TLR-4 in d-galactosamine (D-GalN)-induced rats. Thus, it was indicated that the liver protection activity of limonin occurred via attenuating inflammation and oxidative stress [80].

### 2.6. Other Pharmacological Activity

#### 2.6.1. Neuroprotection

Studies have reported that excessive production of NO could lead to neurotoxicity [81,82]. Studies found that limonin effectively inhibited the influx of calcium and the overproduction of cellular NO and reactive oxygen species (ROS), and had a significant neuroprotective activity on glutamate-induced neurotoxicity in primary cultured rat cortical neurons at the concentration of 0.1 µM. It is worth mentioning that pretreatment with limonin can show a more effective neuroprotective effect, whose mechanism may be found through enhancing the expression of neuroprotective proteins in the stage of cerebral cortex injury [83,84]. In addition, limonin can improve neurodegenerative lesions and cognitive decline by improving the antioxidant capacity of brain tissue [74].

#### 2.6.2. Anti-Osteoporosis

Limonin can increase the concentration of calcium in femur and fifth lumbar in orchidectomized rats, in which the mechanism may be related to promoting bone formation [85]. In addition, studies have found that the loss of ovarian function causes the lack of ovarian-related hormones, which leads to the rapid loss of hormone-related bone and eventually leads to osteoporosis [86]. However, limonin can effectively inhibit the reduction of bone mass and promote the increase of bone mineral density in ovariectomised rats. Moreover, in osteoblastic MC3T3-E1 cells, limonin stimulated alkaline phosphatase (ALP) activity and enhanced the expression of osteoblast differentiation gene markers by regulating extracellular signal-regulated kinase and P38 signals [87]. We believe that limonin is effective against osteoporosis and deserves further study.

#### 2.6.3. Anti-Obesity

Previous research has shown that limonin can reduce the LDL cholesterol in HepG2 cells [88]. Subsequent research had found that nomilin (limonoid) can suppress diet-induced obesity in mice by activating G-protein-coupled receptor (TGR5). Although limonin possessed certain anti-obesity effect, it was not a TGR5 activator; thus, particular mechanisms are remain to be investigated [89]. In addition, limonin inhibited the adipocyte differentiation by reducing the expression of the adipocyte-specific gene (PPARγ2). Limonin can also significantly reduce plasma triglyceride and cholesterol levels in obese mice. Meanwhile, limonin can increase the mRNA levels of acyl-coenzyme A oxidase 1 (Acox1), uncoupling protein 2 (UCP2) and carnitine palmitoyltransferase 1 (CPT1) in liver, which are associated with lipid metabolism [90].

#### 2.6.4. Anti-Allergy

Limonin alleviated 2, 4-dinitrofluorobenzene (DNFB)-induced delayed-type hypersensitivity in mice via inhibiting the activity of adenosine kinase and affecting adenosine metabolism [91]. Moreover, limonin also possesses the effect of treating immunoglobulin E (IgE)-mediated allergies. Limonin exhibited potent inhibitory effect on IgE production by peripheral blood mononuclear cells (PBMCs) and B-cell line from food-allergic pediatric patients, in which the mechanisms may be through inhibiting the transcript expression of ε-germline by PBMCs [92].

#### 2.6.5. Others

In addition, limonin can non-competitively inhibit arginase to increase L-arginine levels, thereby inhibiting the activation of NADPH oxidase in a PKCβII-dependent manner, and blocking nLDL-stimulated VSMC proliferation in a p21Waf1/Cip1 dependent manner [93]. Therefore, it is considered that limonin can treat vascular diseases associated with VSMC proliferation. Meanwhile, limonin can also exert therapeutic effects on mice with idiopathic pulmonary fibrosis by suppressing the expression of iNOS, intercellular cell adhesion molecule-1 (ICAM1), vascular cell adhesion molecule 1 (VCAM1), and COX2, and attenuating oxidative stress in bleomycin-induced lung tissues [94]. Moreover, research had found that limonin possessed a therapeutic effect on experimental gastric ulcer in rats [95]. Interestingly, limonin can inhibit odorant-induced signal transduction pathway (OST) in non-neuronal cells by regulating Ca^2+^ influx and cyclic adenosine monophosphate (cAMP) levels and cAMP response element-binding protein (CREB) phosphorylation, thereby mediating the physiological functions associated with the olfactory receptor [96].

In conclusion, limonin possesses a broad spectrum of pharmacological activities and is used to treat specific diseases (Table 2), indicating that limonin has broad application prospects.

## 3. Toxicity

The research has shown that limonin is toxic to kidney cells. Limonin (50–200 µg/mL) can significantly inhibit the viability of human embryonic kidney cells (HEK-293 cells) in a dose-dependent manner, and can shrink, reduce, and even kill kidney cells in varying degrees at the concentration of 100–200 µg/mL [97]. Meanwhile, limonin also exhibited certain cytotoxicity to T lymphocytic cell line (MOLT-3 cells) and phytohemagglutinin (PHA)-stimulated peripheral blood mononuclear cells (PBMCs) [65]. In addition, limonin possesses obvious cytotoxicity to green monkey kidney cell (COS7 cells) and also has certain cytotoxic effects on HeLa cells [70]. Moreover, limonin extracted from the nearly ripe fruits of *Evodia rutaecarpa* exerted potent cytotoxicity against human promyelocytic leukemia (HL-60) cells and human gastric cancer (N-87) cells [98]. A subsequent study found that limonin extracted from citrus aurantifolia seeds had strong cytotoxicity against L5178Y lymphoma cells [99]. It has also been reported that limonin has a strong inhibitory effect on the production of melanin in B16 melanoma cells, but that it has weak cytotoxicity [34].

Recent studies have found that limonin produces various chromosomal aberrations in hamster lung cells (CHL cells) such as chromosome bridges, chromosome exchange, dicentric, circular chromosomes, and pulverization in the case of 0.025–2.5 mg/mL, and the inhibition rate of limonin on CHL cells was linear. Therefore, it was believed that limonin could cause chromosome distortion in CHL cells and produce genotoxicity [100]. Further research confirmed that the main type of chromosome aberration in CHL cells by limonin is dicentric. Finally, it has been considered that limonin possesses certain genotoxicity and mutagenicity [101].

Mitochondrial permeability transition (MPT) plays an important pathogenic role in mitochondria-mediated hepatocyte injury [102,103]. Limonin has hepatotoxicity. The research has shown that limonin could cause mitochondrial oxidative damage in rats, which results in mitochondrial swelling, MPT pore opening, and the decrease of mitochondrial potential. The hepatotoxic mechanism of limonin may be through inducing MPT, which leads to ATP depletion and cytochrome C release, ultimately triggering cell death signaling pathway [104]. In addition, it has been reported that the volatile components of *Evodia rutaecarpa* mainly contain limonin [105]. The volatile oil of *Evodia rutaecarpa* could cause hepatotoxic damage by decreasing the activity of SOD and GSH-Px and increasing nitric oxide synthase (NOS) activity in serum and liver tissue of mice. Meanwhile, limonin was also detected in the hepatotoxic sites of animals; therefore, it was indicated that limonin might be the material basis of hepatotoxic damage [106,107]. This finding was consistent with the previously reported results of hepatotoxicity induced by limonin.

There are relatively few reports on the toxicity of limonin, but in order to develop this drug safely and effectively, the toxicity of limonin should be further studied and explored. Meanwhile, we also summarized the toxicity mechanism of limonin (Table 3) for the reference of researchers.

## 4. Pharmacokinetics

The study found that limonin was distributed in the duodenum, small intestine, and rectum tissues of rats, among which the drug concentration in the small intestine was the highest, and it reached the peak concentration within 3~4 h after administration. Researchers believe that limonin may exert its therapeutic effect directly in rat intestinal target tissue through local intestinal absorption [108]. Moreover, research confirmed that limonin was absorbed through intestinal diffusion mechanism and absorbed in the whole intestine segment, but that the absorption was poor and saturated. The poor oral absorption of limonin may be due to the co-participation of P-glycoprotein efflux and cytochrome CYP3A4 metabolism [109,110]. Meanwhile, species differences also affected the absorption of limonin. Research found that the time of maximum plasma concentration (T_max_) of limonin was larger in beagle dogs than in rats, and the absorption of limonin was slower in beagle dogs than in rats, but limonin was quickly eliminated in rats and beagle dogs [111,112,113]. In addition, limonin was mainly distributed in the lung tissue of rats, followed by liver, brain, and fat, and less in urine [114].

The oral bioavailability of limonin is very low due to its low solubility and poor permeability [111,115]. Previous studies showed that the oral absorption of limonin was poor, which is mainly due to the active efflux of P-glycoprotein and the first-pass effect of CYP3A4 [116]. The pharmacokinetic study of limonin in rats and beagle dogs indicated that the oral absorption of limonin was poor, and only a small amount of drugs entered the blood. It takes a long time to reach peak blood concentration, but after entering the blood, limonin is gradually metabolized and the elimination half-life is short [3,112,117]. Similarly, findings showed that the blood concentration of limonin in the human body is low and its bioavailability is poor [118]. To enhance the bioavailability of limonin, the study found that when absorption enhancer (verapamil, sodium dodecyl sulfate, polysorbate 80, and borneol) were orally ingested with limonin, they can significantly influence the pharmacokinetic behavior of limonin, among which the absorptive rate of limonin was increased in rat intestine, and the area under concentration–time curve (AUC) and maximum plasma concentration (C_max_) of limonin showed an increasing trend. It was believed that inhibition of P-gp efflux might be an important reason for absorption enhancers promoting the absorption of limonin, which coincides with the previous discovery of poor oral absorption of limonin [119].

In rats, limonin was excreted in the form of prototype and metabolite through urine or feces, which mainly occurs in phase I metabolism, including hydroxylation and hydrogenation [120]. Moreover, phase I metabolites of limonin were found in liver microsomes, urine, and bile of rats, but phase II metabolites were not found, which further confirmed that limonin mainly occurs in phase I metabolism [121]. It is noteworthy that the major excretion pathway of limonin in rats and humans was found to be feces—only a small amount of the drug was absorbed into the blood and metabolized by the liver, and a small amount of the drug was excreted through the urine [122]. In human liver microsomes (HLMS), the major metabolic pathways of limonin were found to be reduction at C-16 carbonyl, hydroxylation, and reaction of glycine with reduction of limonin. Moreover, CYP3A4 and CYP2D6 were found to play an important role in the glycosylation and isomerization of limonin during metabolism [123,124]. In addition, the major metabolic pathways of limonin in the human body were found to be reduction, hydrolysis and methylation, and it was found that limonin could be more widely metabolized by human intestinal bacteria through the above metabolic pathways [125]. Limonin partially inhibited the activities of human cytochrome P450 (CYP) isoenzymes such as CYP3A4, CYP19 and CYP1B1, in which limonin irreversibly inactivates CYP3A4 [78,126,127]. However, CYP3A4 was also found to be a key enzyme in the synthesis of limonin metabolites (electrophilic *cis*-enedial intermediate) in mice and human liver microsomes. Thus, researchers speculated that the mechanism of limonin-induced enzyme inactivation might be due to the combination of related enzymes with *cis*-enedial intermediate of limonin [128].

The induction or inhibition of CYP450 enzyme isoforms was considered to be the key cause of clinical drug interaction [129]. Among them, limonin could speed up the metabolism of some drugs and reduce their efficacy by inducing the expression of CYP1A2 [130]. Therefore, it was suggested that the combination of limonin with these drugs should be avoided in clinical use. Certain diseases and gender differences also affect the pharmacokinetic characteristics of limonin. One study found that the AUC, C_max_ and T_max_ of limonin decreased and caused T_1/2_ delay in headache model rats [131]. There were marked gender differences in pharmacokinetics of limonin either orally or intravenously. The C_max_ and AUC of limonin in female rats were found to be much higher than those in males. Further studies revealed that the gender differences of CYP3A2 and CYP2C11 in liver microsomes of rats might be the main reason for gender differences in pharmacokinetics [132,133]. Furthermore, we also summarized the relevant pharmacokinetic parameters of limonin in animals in Table 4, in order to provide reference for the preclinical pharmacokinetic study of limonin.

## 5. Conclusions and Future Perspectives

Limonin is abundant in plant resources and widely exists in many traditional medicines that possess high medicinal value, especially in anti-cancer, anti-inflammatory and analgesic, anti-bacterial, and anti-viral treatment, having great clinical application potential. In vivo and in vitro research showed that limonin can regulate the expression of related genes and proteins, including BCL2/Bax, MCP-1, p53, p21, miR-216a-3p, advanced glycation end products (AGEs), TNF-α, iNOS, IL-1β, IL-2, IFN-γ, and HIV-1 gag. In addition, limonin also affects Wnt5/beta-catenin, TLR4/NF-κB, OST, and TLR signaling pathways. This review summarized the mechanism of limonin in the treatment of specific diseases and the toxicity mechanism of limonin. Therefore, in the future, we can further clarify the existing issues on the basis of the aforementioned research.

On the basis of the literature, we found that most researchers believe that limonin does have antioxidant activity. However, a few researchers have questioned whether limonin could be regarded as an antioxidant or not. Among them, the different conclusions may be attributable to the difference in the special structure and dosage of limonin. Therefore, more work should be devoted to exploring the specific antioxidant mechanism of limonin at the molecular level, as well as further evaluating the potential medicinal value.

Limonin exerts hepatotoxicity and hepatoprotective activity. Through summarizing and analyzing the literature, we believe that this is mainly related to the time and concentration of limonin administration, whereas long-term and high-dose administration may lead to hepatotoxicity. However, up until now, we have not found any research reports on reducing the toxicity of limonin. Moreover, related target-organ toxicity evaluations are also lacking. In order to develop and utilize limonin safely and effectively, it is necessary to pay more attention to the in-depth study of the toxicity mechanism of limonin towards target-organ, and to explore the ways in which its toxicity can be reduced in future.

Pharmacokinetic studies showed that limonin had low solubility, poor oral absorption, and low bioavailability, mainly due to the insoluble chemical structure of limonin and its ability to activate the activity of p-gp. In addition, we also found that the location and group of limonin substituents are the key factors affecting pharmacological activity and bioavailability. The substitution of C-7 position of limonin has important structural characteristics, and new structural derivatives can significantly enhance anti-inflammatory, analgesic, and antimicrobial activities, and show higher water solubility to improve bioavailability. Therefore, we believe that the structural modification of limonin will be the focus of future research.

In recent years, limonin has attracted considerable interest in the medicinal chemistry society, owing to its promising multiple pharmacological activities and intriguing structure. However, its imprecise mechanism of action, limited water solubility, poor oral bioavailability, and complex toxicity have greatly hindered its clinical potential. Hence, to improve such problems, studies on the combination of limonin with other drugs have been constructed to enhance therapeutic effects and bioavailability. This has been confirmed in terms of anticancer effect and the promotion of intestinal absorption. Nevertheless, to advance limonin into viable clinical therapies, there are several new directions for future studies in the area: (1) Although limonin has already been proven to possess multiple pharmacological activities in in vitro and in vivo studies, its specific mechanism of biological activity has not yet been fully determined, such as its mechanism of antioxidant action. Hence, it is important to further explore the mechanism of biological activity at the molecular level. (2) Aside from its activities, increasing research has found its potential toxicity. Consequently, it is necessary to design a strategy to balance the pharmacological effects and toxicity of limonin. First, more systematic studies on the effects of dosage on pharmacological activity and toxicity should be constructed. Second, more research is also needed to lessen its side effects and toxicity. (3) Structural modification is a promising method for obtaining some limonin derivatives with good therapeutic effect and high bioavailability. Therefore, the rational design of new limonin derivatives is of great significance for the development of new drugs in the future.

## Figures and Tables

**Table 1:** Plants containing limonin.

Family	Plant Materials	Plant Species	Plant Part	Ref.
Rutaceae	*Evodia rutaecarpa*	*Euodia rutaecarpa* (Juss.) Benth.; *Euodia rutaecarpa* (Juss.) Benth. var. *officinalis* (Dode) Huang; *Euodia rutaecarpa* (Juss.) Benth. var. *bodinieri* (Dode) Huang	Fruits	[1]
	*Cortex dictamni*	*Dictamnus dasycarpus* Turcz.	Root bark	[3]
	*Cortex chinensis phellodendri*	*Phellodendron chinense* Schneid.	Stem bark	[4]
	Bergamot	*Citrus bergamia,* C.b	Fruits	[5]
	*Aurantii fructus immaturus*	*Citrus aurantium* L.; *Citrus sinensis* osbeck	Fruits	[6]
	*Citri reticulatae pericarpium*	*Citrus reticulata* Blanco.	Peels	[7]
	Citrus	*Citrus reticulata* Blanco.	Fruits	[8]
	Pummelo	*Citrus maxima* (Burm) Merr.	Seeds	[10]
	Buntan fruit	*Citrus grandis* Osbeck	Fruits	[11]
Meliacea	*Toosendan fructus*	*Melia toosendan* Sieb. et Zucc.	Fruits	[12]
Ranunculaceae	*Coptidis rhizoma*	*Coptis chinensis* Franch.; *Coptis deltoidea* C. Y. Cheng et Hsiao; *Coptis teeta* Wall	Rhizomes and roots	[2]

**Table 2:** Biological and pharmacological activities of limonin—in vitro and in vivo studies summary.

Pharmacological Effects	Detail	Cell Lines/Model	Dosage of Administration	Application	Ref.
Anticancer activity	Activation of endogenous apoptosis pathway	SW480 cells	IC50 = 54.74 μM	In vitro	[15]
	Expression of apoptosis-related proteins promotes apoptosis of tumor cells	HCT-15 and SNU449 cells	4.25, 42.5, and 425 nmol/mL	In vitro	[16]
	Inducing apoptosis	AOM-injected rats	200 mg/kg	In vivo	[19]
	Decreased expression of c-Myc and MCP-1mRNA	Apc-mutant Min mice	250 and 500 ppm	In vivo	[20]
	Inhibiting the growth of tumor cells	SMMC-7721 cells	IC50 = 24.42 µg/mL	In vitro	[21]
	Inducing apoptosis	HepG2 cells	IC50 = 60 μM	In vitro	[22]
	Inhibiting the activity of hexokinase-2	Hepatocellular carcinoma cells	25, 50, and 100 μM	In vitro	[23]
	Through the induction of xenobiotic enzymes	Aflatoxin B1 induced liver cancer rat	50 mg/kg	In vivo	[24]
	Inducing apoptosis	MCF-7 and MDA-MB-231 cells	1, 5, and 10 μM	In vitro	[28]
	Inhibiting the expression of p53 and p21 and activating the endogenous pathway	Panc-28 cells	IC50 = 42.4 μM	In vitro	[30]
	Inhibition of Wnt5/β-catenin pathway	IOMM-Lee and CH157MN cells	25 μM	In vitro	[31]
	Inhibiting tumor proliferation	Lung cancer A549 cells	IC50 = 82.5 μM	In vitro	[32]
	Inhibiting the growth of tumor cells and promoting apoptosis	HeLa cells	50 μM	In vitro	[33]
	Inhibiting the efflux of P-GP substrate rhodamine 123	Caco-2 and CEM/ADR5000 cells	20 μM	In vitro	[35]
	Increasing the expression of miR-216a-3p	MCF-7 and MDA-MB-231 cells	5, 10, and 20 µM	In vitro	[36]
	Promoting the nuclear-cytoplasmic translocation of YAP	HeLa and Cervical carcinoma cell lines (C33A) cells	5, 10, and 20 µM	In vitro	[37]
Anti-inflammatory and analgesic activity	Inhibiting the proliferation of CD4^+^ T-cells	Transgenic mice	200 mg/kg	In vivo	[38]
	Inhibiting the activity of p38 MAP kinase in cells	Human aortic smooth muscle cells	12.5, 25, and 50 µM	In vitro	[39]
	Decreasing serum AGEs, TNF- α, and MDA levels	Male albino rats	50 mg/kg	In vivo	[40]
	Inhibition of NO production	RAW264.7 macrophages	IC50 = 231.4 µM	In vitro	[41]
	Decreasing the expression of *iNOS* gene	Wistar rats hepatocytes	IC50 = 16 µM	In vitro	[42]
	Inhibiting activation of NF-κB p65	C57BL/6 mice	50 mg/kg	In vivo	[44]
	Regulation of TLR4/NF-κB pathway	ALI mice	10 mg/kg (ip)	In vivo	[45]
	Decreasing the mRNA expression level of IL-1 β, neutrophil chemoattractant 1 (CINC-1) and mucin gene (MUC5B, MUC5AC)	Airway inflammation Wistar rat	20 µM (6mL)	In vivo	[46]
	Decreasing the mRNA expression of IL-2, IL-17a, TNF- α, and IFN- γ, and inhibiting the growth of T lymphocytes	BALB/c mice and CD3^+^ T cells	5, 10, and 20 mg/kg, and 0.1, 1, 10, and 100 uM, respectively	In vivo and In vitro	[47]
	Regulating metabolic pathways	NBP male Wistar rats	3.402 g/kg	In vivo	[48]
Antibacterial, antiviral and anti-insect activities	Inhibiting proliferation	Xanthomonas sp.SK12; X. campestris pv. Compestris KC94-17-XCC; X. oryzae pv. oryzae KX019-XCO; X. campestris pv. Vesicatoria YK93-4-XCV;	MIC = 15.62, 31.25, 31.25, and 62.5 µg/mL	In vitro	[57]
	Combination with DapF and MurA	Acinetobacter baumannii	25, 50, 75, and 100 µL	In vitro	[58]
	Inhibition of biofilm formation and TTSS	E. Coli O157:H7	6.25, 12.5, 25, 50, and 100 µg/mL	In vitro	[60]
	Inhibiting cell–cell signaling and biofilm formation	*Escherichia coli*, *Vibrio harveyi*	6.25, 12.5, 50, and 100 mg/mL	In vitro	[61]
	Inhibition of HIV-1 protease activity	HIV-1	EC50 = 60.0 µM	In vitro	[62]
	Anti-HSV activities	HSV-1 and HSV-2	100 µg/mL	In vitro	[64]
	Inhibiting the expression of HTLV-1 Tax/rex and HIV-1 gag	HTLV-1 infected cells; HIV-1 infected cells	IC50 = 1.07 and 0.92 µg/mL, respectively	In vitro	[65]
	Inhibition of growth and development	New ring-stage *P. falciparum* parasites	IC50 = 2.7 µM	In vitro	[66]
	Nematocidal toxicity	*Meloidogyne incognita*	LC50 = 197.37 µg/mL	In vitro	[67]
	Antiparasitic activity	Mice harboring *Schistosoma mansoni*	50 and 100 mg/kg	in vivo	[68]
Antioxidant activity	Reducing the accumulation of fatty acid oxidation products	Syrian Golden Hamsters plasma	10 µM	In vitro	[73]
	Increasing plasma antioxidant status	Orchidectomized male rats	200 mg/kg	In vivo	[85]
	Reducing MDA and GSH-Px levels, increasing SOD, GSH-Px activity and T-AOC capability	Natural aging SD rats	50 and 150 mg/kg	In vivo	[74]
Liver protection activity	Down-regulation of TLR signaling pathway	(I/R) liver injury rat	100 mg/kg	In vivo	[79]
	Reducing inflammation and oxidative stress	D-GalN- induced liver injury rat	50 and 100 mg/kg	In vivo	[80]
Other pharmacological activity	Enhancing the expression of neuroprotective proteins	Rat cortical cells	0.05 and 0.1 µM	In vitro	[84]
	Protecting nerve cells	Natural apolexis SD rats	50 and 150 mg/kg	In vivo	[74]
	Preserving bone calcium concentration and increasing antioxidant status	Orchidectomized male rats	200 mg/kg	In vivo	[85]
	Increasing bone mineral density and osteoblast differentiation	Ovariectomised rats and MC3T3-E1 cells	250 mg/kg, and 5, 10, 20, and 40 µM, respectively	In vivo and in vitro	[87]
	Inhibiting the differentiation of adipocytes and increase the level of lipid metabolism genes	Mouse Preadipocyte (3T3-L1) cells and Diet-induced obese mice	30, 50, and 100 mg/mL, and 100 mg/kg, respectively	In vitro and in vivo	[90]
	Inhibition of adeno-kinase activity	Balb/c inbred mice	5, 10, and 20 mg/kg	In vivo	[91]
	Mediated IgE suppression	Human B-cell line (U266) cells	1.25, 2.15, 5, 10, and 20 µM	In vitro	[92]
	Inhibition of arginase activity	Rat aortic smooth muscle cells	25 and 50 µM	In vitro	[93]
	Reducing inflammation and oxidative stress	Pulmonary fibrosis mice and MLE-12 cells	25 and 50 mg/kg, and 3, 10, and 30 µM, respectively	In vivo and in vitro	[94]
	Inhibiting OST pathway	T lymphocytic line (3T3-L1) cells	200, 400, 600, and 800 µM	In vitro	[96]

**Table 3:** Toxicity of limonin.

Activity/Mechanism(s) of Action	Cell Lines/Model	Dosage of Administration	Application	Ref.
Inhibition of cell viability	HEK-293 cells	25–200 µg/mL	In vitro	[97]
Inhibiting the metabolic activity of cell	MOLT-3 cells and PHA stimulated PBMCs	1 ng/mL to 1 mg/mL	In vitro	[65]
Inhibiting cell growth	COS7 and HeLa cells	IC50 = 35.0 and 132.1 μΜ, respectively	In vitro	[70]
Inhibition of cell viability	L5178Y lymphoma cells	IC50 = 8.5 µg/mL	In vitro	[99]
Inhibition of cell viability	B16 melanoma cells	10, 30, and 100 μM	In vitro	[34]
Chromosome aberration	CHL cells	IC50 = 2.5 mg/mL	In vitro	[100]
Mitochondria oxidative damage	Male SD rats	44.8, 89.6, and 179.2 mg/kg	In vivo	[104]

**Table 4:** Pharmacokinetic parameters of limonin in animals after oral administration.

Inclusion of Drug Components	Oral Dosage	Animal Model	Pharmacokinetic Parameters of Limonin	Ref.
Limonin	19 mg/kg	Wistar rats	C_max_ = 67.8 ± 46.2 ng/L, T_max_ = 0.3 ± 0.2 h, T_1/2_ = 4.9 ± 1.9 h	[112]
Limonin	10 mg/kg	Beagle dogs	C_max_ = 28.68 ± 12.99 ng/L, T_max_ = 2.17 ± 0.76 h, T_1/2_ = 6.47 ± 2.08 h, AUC_0–∞_ = 128.30 ± 65.34 ng h/mL	[113]
Limonin	30 mg/kg	Beagle dogs	C_max_ = 34.78 ± 13.36 ng/L, T_max_ = 17 ± 1.85 h, T_1/2_ = 2.48 ± 0.12 h	[117]
Cortex Dictamni extract	0.424 g/kg (38 mg/kg of limonin)	Male Sprague–Dawley rat	C_max_ = 419 ± 97.4 ng/L, T_max_ = 0.78 ± 0.17 h, T_1/2_ = 8.7 ± 2.15 h, AUC_0–∞_ = 1559 ng h/mL	[3]
Wu-Zhu-Yu decoction	6.67 g/kg	Normal Male Sprague–Dawley rats	C_max_ = 630.9 ± 446.0 ng/L, T_max_ = 1.76 ± 2.16 h, T_1/2_ = 34.59 ± 6.10 h	[131]
Wu-Zhu-Yu decoction	6.67 g/kg	Headache male Sprague–Dawley rats	C_max_ = 550.8 ± 319.0 ng/L, T_max_ = 0.73 ± 0.37 h, T_1/2_ = 49.32 ± 23.63 h	[131]
Limonin	11.8 mg/kg	Male and female Sprague–Dawley rats	C_max_ = 41.61 ± 7.48 ng/L, T_max_ = 0.63 ± 0.14 h, T_1/2_ = 4.25 ± 1.72 h	[134]

C_max_: maximum plasma concentration; T_max_: the time of maximum plasma concentration; AUC_0–∞_: the area under the plasma concentration time curve from 0 to ∞; T_1/2_: the elimination half-life.
